# Hand-Rearing of Three Lesser Flamingo Chicks (*Phoeniconaias minor*)

**DOI:** 10.3390/ani10081251

**Published:** 2020-07-23

**Authors:** Letizia Fiorucci, Francesco Grande, Roberto Macrelli, Petra Schnitzer, Lorenzo Crosta

**Affiliations:** 1 Facultad de Veterinaria, Universidad de Las Palmas de Gran Canaria, Arucas, 35416 Las Palmas de Gran Canaria, Spain; 2 Loro Parque Fundación, Avenida Loro Parque, 38400 Puerto de la Cruz, Spain; fragrande@alice.it; 3 Dipartimento di Scienze Pure e Applicate, Università di Urbino, 61029 Urbino, Italy; macroberto@libero.it; 4 Avian, Reptile&Exotic Pet Hospital, Sydney School of Veterinary Science, The University of Sydney, Camden 2570, Australia; petra_schnitzer@yahoo.com (P.S.); lorenzo.crosta@sydney.edu.au (L.C.)

**Keywords:** hand-rearing, diet, growth index, lesser flamingo (*Phoeniconaias minor*), spirulina (*Spirulina platensis*)

## Abstract

**Simple Summary:**

Like many other colonial nesting waterbirds, all six flamingo species are considered of conservation concern because of their dependence on a limited number of wetlands, particularly for breeding. Population decreases of some species have been linked to changes in the ecosystem. Specifically, according to the International Union for Conservation of Nature (IUCN), the lesser flamingo (*Phoeniconaias minor*) population is declining and near threatened. The ability to hand-rear nestlings of this endangered species and return them to the wild is, therefore, an important aspect of the conservation of the lesser flamingo and of flamingos in general. Hand-rearing of abandoned chicks is recommended as a conservation tool to limit mortality and to bolster the population at specific colonies. When adults are not able to rear chicks, chicks must be offered a diet that allows them to maintain adequate growth and development. Successful hand-rearing is based on a formula that meets the nutritional needs of the chicks as they develop. The aim of this study is to describe the diet composition, dietary intake, feeding protocols, and growth index of three lesser flamingo chicks hand-reared with the diet described in the paper to share important data useful for the conservation of the populations in the wild. All the aforementioned parameters were recorded daily, from hatching to 2 months of age.

**Abstract:**

There are few published studies regarding lesser flamingo (*Phoeniconaias minor*) reproduction, crop milk composition, and hand-rearing under human care. Between the end of June and the beginning of August of 2017, three eggs were laid in a group of 29 lesser flamingos kept under human care. Two eggs and one chick were abandoned by the parents, and three chicks were hand-reared. This report describes diet composition, dietary intake, feeding protocols, and growth index, from the first day to 60 days after hatching, for three lesser flamingo chicks.

## 1. Introduction

There are only six existing flamingo species. They all belong to the family Phoenicopteridae, within the order Phoenicopteriformes. Flamingos are large waterbirds with long necks and legs and unique down curved bills adapted to filter-feeding. They are widely distributed in all regions except Australasia and the Antarctic. Flamingos tend to inhabit shallow waterbodies, normally saline, brackish, or alkaline. They may live in coastal or inland water bodies, and their altitude distribution is also wide, ranging from sea-level up to around 5000 m. The lesser flamingo (*Phoeniconaias minor)* is the flagship species of the saline wetlands of Africa and India and is a specialized feeder subsisting on microscopic cyanobacteria and algae. The *in situ* preferred food organisms consist of filamentous cyanobacteria, mainly *Spirulina platensis*, as well as benthic diatoms [1,2]. Many studies showed that temporal fluctuations in lesser flamingo populations are related to the changes in the abundance and diversity of phytoplankton species [3,4,5,6,7,8,9,10,11]. The occurrence of *Spirulina* spp. was associated with the increase in the abundance of lesser flamingos. It was observed that changes in the lesser flamingo numbers were influenced by the changes in the abundance and availability of their preferred food [3,4,5,6,7,8,9,10,11]. All flamingo species (Phoenicopteridae) feed their nestlings using crop secretions, called crop milk, originating from esophageal glands, for up to six months [12,13,14,15,16,17]. As seen in several species of Columbiformes and some penguins, the composition of crop milk varies with stage of development, but it has not been examined in detail for lesser flamingos [12,13,14,15,16,17]. When adults are not able to rear chicks, chicks must be offered a diet that allows them to maintain adequate growth and development. Successful hand-rearing is based on a formula that meets the nutritional needs of the chicks as they develop. In addition, it is important that the formula be readily digested by chicks, and its consistency must be appropriate for delivery with a feeding tube [12,13,14,15,16,17]. This short report describes diet composition, dietary intake, and feeding protocols for hand-rearing lesser flamingo nestlings.

## 2. Materials and Methods

### 2.1. Animal Collection

Between the end of June and the beginning of August of 2017, three eggs were laid in a group of 29 lesser flamingos (ratio 23.6) kept under human care. The size of eggs was recorded: the length ranged from 88 (egg 1) to 84 mm (eggs 2 and 3), the width was 50 mm for all three eggs, and the weight ranged from 94.0 (egg 1) to 91.7 (egg 2) and finally 90.9 g (egg 3). Two eggs were naturally incubated by parents; the last one was abandoned and maintained in an artificial incubator (temperature: 37.2 °C, humidity: 55%) for 28 days until it hatched spontaneously, with no need for assisted hatching. Concerning the two naturally incubated eggs, a chick was born from the first egg laid, which was initially raised by the parents and then abandoned after just 2 days. The second egg was abandoned at the time of hatching. The abandonment of the nest may have been due to the noise of the maintenance work from an enclosure adjacent to the nesting area. Two eggs and one chick were abandoned by parents and three chicks were hand-reared. The chicks remained in the hatcher until they looked dry and healthy. After this, the chicks were placed into a bin of an appropriate size (30 × 20 × 20 cm) and put in a brooder specifically designed for birds (Camera calda VPL, 36063 Vicenza, Italy). Temperature was maintained at 34 °C and humidity at 47%.

During the first 24 h of hand-rearing, the chicks were fed only Ringer’s lactate solution, a total of 6 mL each, in order to keep them hydrated. The allantoises were treated with disinfectant for two days, and the first feeding occurred 24 h post-hatch. The three chicks were hand-reared, with the feeding formula described in the next paragraph, two of them since the first day after hatch. All other aspects of general husbandry followed the published Flamingo Husbandry Guidelines [18].

### 2.2. Diet (Formula)

The formula used was based on a modification of the formula initially used by Sea World California and Fort Worth Zoo, Texas, enriched with *Spirulina platensis* [16,17,18]. The hand feeding formula was composed of 400 mL natural mineral water, 75 g capelin (*Mallotus villosus*) fillets, 75 g *Krill pacifica* (Ruto Frozen Fishfood, 3417 XH Montfoort, The Netherlands), 20 g human baby organic rice flour (Crema di Riso Crescendo Coop, 40033 Casalecchio di Reno, Italy), 1 medium sized hard-boiled hen’s egg yolk, (18 g), 1 (0.75 g) Vita-Zu Large Bird Tablet 5M23 (Mazuri^®^. PMI Nutrition International, Brentwood, 90049, U.S.A), 0.4 g *Spirulina platensis* (Arkocapsule^®^, Arkopharma, 18039 Ventimiglia, France), and 0.125 g calcium carbonate (CaCO_3_—Calcium Sandoz, Sandoz Pharmaceuticals GmbH, 83607 Holzkirchen, Germany). The formula preparation followed a specific step-by-step protocol: the rice flour, hard-boiled hen’s egg yolk, calcium carbonate, vitamin tablet, and 200 mL water were mixed into a liquid form and put in a bowl. Then, the capelin fillets, the *Krill pacifica*, the *Spirulina platensis*, and 200 mL water were mixed in separate bowl, filtered with a strainer to get rid of any coarse particles, and mixed with the previous liquid blend. This final formula could be stored in a fridge for up to 12 h prior to being warmed to 39 °C and fed to the chicks.

### 2.3. Statistical Analyses

Descriptive statistics was employed, and Medcalc, Version 11.6.0.0 (MedCalc Software Ltd, Acacialaan 22, 8400 Ostend, Belgium) was used to analyze the data.

## 3. Results

Days 1–4: Chicks showed pinkish bills and legs, prominent egg teeth, and stood with difficulty at day 2. During this period, feces were orange. Chicks were fed a 1:2 (formula/water) dilution of the original feeding formula. Every morning the chicks were weighed, and the total amount of feeding formula to be fed was determined prior to the first feeding of the day, based on weight gains. The formula amount per session was no more than 12% of chick body mass (BM).

Feedings were spaced at 3-h intervals for a total of six feedings/day, between 5 a.m. and 11 p.m. Amounts fed were gradually increased (1 to 20 mL) to maintain a steadily increasing weight curve. Birds were fed using syringes with different sizes depending on the bird’s size.

Days 5–14: Chicks showed greyish bills and legs. The egg teeth were still visible, and the birds could stand easily at day 5, walking actively from day 7. Feces became greenish and remained this color for the entire period. Chicks were fed a 1:1 (formula/water) diluted mixture, and the total amount of diluted food was 10–12% of BM. Feedings were spaced at 4-h intervals for a total of five feedings/day from 7 a.m. to 11 p.m.

Days 15–24: Chicks showed black legs and slightly curved bills. They were fed full-strength formula, still between 10% and 12% of BW, 4 feedings/day (7 a.m., 11 a.m., 3 p.m., and 7 p.m.), and were taken out to exercise, sun, and bath once daily at least for 1 h. The average external temperature was 31.5 °C. In addition, a bowl with 250 mL of formula was available for each chick at all times.

Days 25–31: Chicks were tube-fed three times daily (7 a.m., 1 p.m., 7 p.m., from 60 to 80 mL, 10–12% of BM) and exercised outdoors, with access to a shallow pool. In addition, a bowl with 500 mL of formula was available for each chick at all times. Primary feathers started to emerge.

Days 32–45: Chicks were fed with formula plus pellets (Flamingo Complete Diet, Mazuri^®^, PMI Nutrition International, Brentwood, CA, USA). At the beginning, the pellets were ground, in order to have a transition to the adult flamingo diet. Tube-feeding decreased to two times daily (8 a.m. and 8 p.m.) as long as the birds continued to gain weight, and chicks had access to the pool at all times. Only 80 mL/feeding of formula was offered to avoid excessive dilatation of the crop and to encourage self-feeding from the bowl.

Days 46–60: Tube feeding dropped to up to once per day (8 a.m.). Chicks had access to a food dispenser with adult food breeder pellets. The chicks shared the enclosure with the adults during the day and were separated for the night. Diurnal observation was performed, and no social/behavioral problems were noted. Chicks showed full plumage (from day 46), and they started to fly. Weaning between days 46 to 60 worked well.

**Figure 1:**
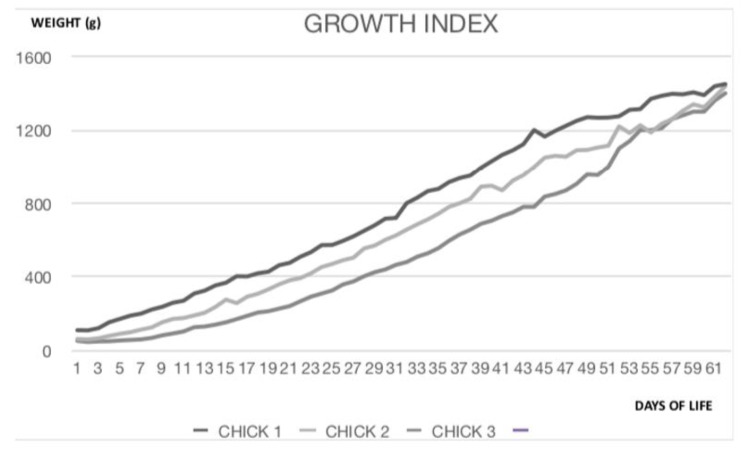
Daily growth data (for the first 60 days after birth) of lesser flamingo chicks of the present study.

Daily growth data (for the first 60 days after birth) of the three lesser flamingo chicks are shown in Figure 1.

The hand feeding formula described in this study contains as dry matter 38.8% fat and 47.2% protein. The nutrient composition of the present diet is comparable with the American flamingo crop milk composition [16,17,18,19,20,21,22,23]. The formula used in the present study had 614 kcal/100 g. The early stage hand-rearing formulas (on a dry matter basis) reported in the literature contain roughly 40% protein and 20% fat, averaging between 25% and 35% solids [16,17,18,19,20,21,22,23]. This is comparable to the composition of flamingo crop milk [8,17] containing 58% fat and 35% protein (dry matter basis), with a solids content of approximately 10% to 20%. However, artificial diets are substantially different from the secretions provided by the adults, as well as for the use of carbohydrates, usually baby cereal, as chicks probably do not have the enzymes required to process them [16,17,18,19,20,21,22,23].

**Figure 2:**
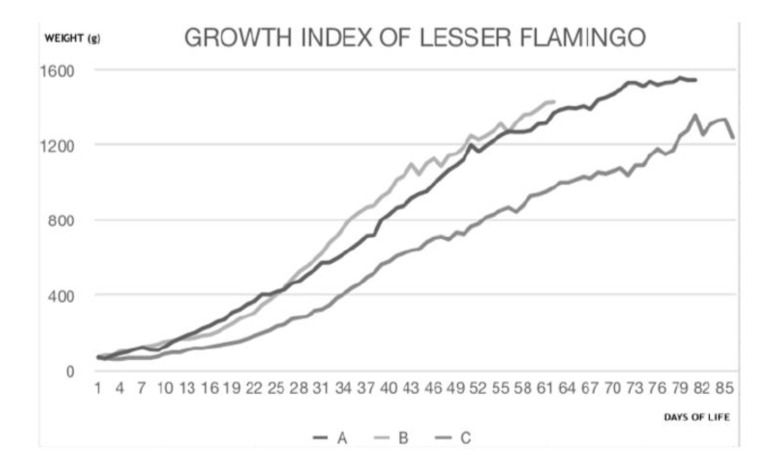
**A**: The present study; **B**: SeaWorld California; **C**: Fort Worth Zoo, Texas. Daily growth data of the three lesser flamingo chicks in the present study correspond to data previously described by Burch and Gailband (2000) at a SeaWorld institution and by Ward (2001) at Fort Worth Zoo [16,17].

In this study, growth patterns between the lesser flamingo and grater flamingo [16,17,18,19,20,21,22,23] seem to be similar. Daily growth data of the three lesser flamingo chicks in the present study compared to previous studies are shown in Figure 2.

## 4. Discussion

To the authors’ knowledge, no analyses of amino acids in flamingo crop milk have been reported; however, the nutrient composition is known, and the present formulation seems to be more similar compared to previous formulations. Details of nutritional requirements for specific fatty acids, vitamins, and/or minerals of lesser flamingos may further improve this formulation [16,17,18,19,20,21,22,23].

The fat content of the formula falls toward the lower end of the range for that reported for crop milk [16,17,18,19,20,21,22,23]. No other sources of fat were added to the formula to increase the percentage of fat.

The growth of the chicks was normal, and there was apparently no issue with the development of the feathers, beaks, or nails. On the second day after hatching, all the chicks showed a physiological weight loss of about 10%; we presume that this is a normal response to the end of the absorption of the yolk. From the third day onwards, the weight increase was progressive and constant at ~10% body weight per day. During the first period (days 1–4), feces were orange, then they became green and remained this color throughout hand-rearing. Furthermore, no problems associated with diet preparation, administration, or animal responses were reported.

In previous studies, differences between Caribbean and Chilean flamingo chick growth patterns were noted. Species differences in metabolism may underlie these observations; Chilean flamingo chicks can tolerate cooler temperatures, and a slower growth rate may indicate a lower metabolism. Chilean flamingos have been found to have longer gastrointestinal tracts, with larger caeca, which may also indicate metabolic discrepancies between these two species [16,17,18,19,20,21,22,23].

*Spirulina platensis* is the main food source for these birds in the wild [24]. These microalgae are commonly used by humans and animals as a source of protein. *Spirulina platensis* is said to have several properties, such as good palatability, lack of toxicity, easy digestion, and antioxidant activity, as well as hypocholesterolemic, anticancer, immunostimulant, anti-inflammatory, and antiviral characteristics, among others. Several studies have been conducted to verify the possible benefits of spirulina, and some properties have been verified [25,26,27,28,29]. Moreover, it is known that, in the wild, an adult lesser flamingo requires a daily supply of about 70 g∙d−1 dry mass of *Spirulina* spp. [24]. For this reason, the authors decided to supplement the diet of growing flamingos with *Spirulina platensis*, instead of using carotenes or other substances, which may also have important activity as antioxidant or allow a proper coloration of the plumage in flamingos. According to Ross and Dominy (1990) [27], the addition of 1.5% to 12% of *Spirulina platensis* into the diets for broilers can replace other protein sources, especially soybean meal, with satisfactory growth rates and feed efficiency [27]. However, the optimal levels for using this alga as a substitute source of the conventional protein in a diet are still controversial [25,26,27]. Since, in the literature, there is no mention of the use of *Spirulina* spp. as the only supplement for hand feeding the lesser flamingo, the authors decided to include a low percentage of *Spirulina platensis* (0.1%) in the hand-rearing diet of the lesser flamingo chicks. This amount is, however, consistent with the use of *Spirulina* spp. in the diets of other animals [30]. Thus, the authors consider it important to focus on this point as further studies are necessary in order to evaluate the nutritional value of *Spirulina* spp. for these animals to evaluate whether it could be used as the main protein source of the whole diet.

## 5. Conclusions

This study devised another perfectly acceptable diet formula that can be used to successfully raise flamingo nestlings from hatching to independence. Moreover, it mimics the nutrient content of flamingo crop milk, since there was no analysis of flamingo crop milk reported in the literature. The hand-rearing of the three nestlings was a completely successful experience, since all of them remain in good health to the present day. Studies on the rearing by hand of such fragile species as minor flamingos can provide useful data for the conservation of the species, as the optimal conditions for food supply and reproduction are undermined by climate change [10,31]. The goal for further studies could be evaluating features of the gastrointestinal tract, metabolism, and crop milk composition of the lesser flamingo and developing a formula that mimics the nutrient content of actual flamingo crop milk.
